# Large-Scale Analysis of Fitness Cost of *tet*(X4)-Positive Plasmids in *Escherichia coli*

**DOI:** 10.3389/fcimb.2022.798802

**Published:** 2022-06-03

**Authors:** Feifei Tang, Wenhui Cai, Lijie Jiang, Zhiqiang Wang, Yuan Liu

**Affiliations:** ^1^College of Veterinary Medicine, Yangzhou University, Yangzhou, China; ^2^Jiangsu Co-innovation Center for Prevention and Control of Important Animal Infectious Diseases and Zoonoses, Yangzhou University, Yangzhou, China; ^3^Joint International Research Laboratory of Agriculture and Agri-Product Safety, the Ministry of Education of China, Yangzhou University, Yangzhou, China; ^4^Institute of Comparative Medicine, Yangzhou University, Yangzhou, China

**Keywords:** tigecycline, *tet*(X4), plasmids, fitness cost, bacteria, large-scale analysis

## Abstract

Tigecycline is one of important antimicrobial agents for the treatment of infections caused by multidrug-resistant (MDR) Gram-negative bacteria. However, the emergence and prevalence of plasmid-mediated tigecycline resistance gene *tet*(X4) are threatening human and animal health. Fitness cost elicited by resistance plasmids is a key factor affecting the maintenance and transmission of antibiotic resistance genes (ARGs) in the host. A comparative analysis of the fitness cost of different types of *tet*(X4)-positive plasmids is helpful to understand and predict the prevalence of dominant plasmids. In this study, we performed a large-scale analysis of fitness cost of *tet*(X4)-positive plasmids origin from clinical isolates. These plasmids were successfully electroporated into a reference strain *Escherichia coli* TOP10, and a series of transformants carrying the *tet*(X) gene were obtained. The effects of *tet*(X4)-positive plasmids on the growth rate, plasmid stability, relative fitness, biofilm formation, and virulence in a *Galleria mellonella* model were evaluated. Consequently, we found that these plasmids resulted in varying degrees of fitness cost on TOP10, including delayed bacterial growth and attenuated virulence. Out of these plasmids, *tet*(X4)-harboring IncFII plasmids showed the lowest fitness cost on the host. Furthermore, by means of experimental evolution in the presence of commonly used drugs in clinic, the fitness cost of *tet*(X4)-positive plasmids was substantially alleviated, accompanied by increased plasmid stability. Collectively, our data reveal the differential fitness cost caused by different types of *tet*(X4)-positive plasmids and suggest that the wide use of tetracycline antibiotics may promote the evolution of plasmids.

## Introduction

Antibiotic drugs have offered a powerful weapon in the fight against bacterial infections over the past decades. However, the massive use of antibiotics has led to the emergence of multidrug-resistant (MDR), extensively drug-resistant (XDR), and pandrug-resistant (PDR) bacteria, which severely diminishes the efficacy of clinically relevant antibiotics and constitutes a global public health problem (Zhong et al., 2014; Fang et al., 2020). Tigecycline is a broad-spectrum antibacterial drug of the glycyltetracyclines and has great antibacterial activity against hard-to-treat drug-resistant pathogens such as methicillin-resistant *Staphylococcus aureus* and carbapenem-resistant pathogens (Labreck and Merrell, 2020; Xu et al., 2021a). In clinic, tigecycline is widely used in the treatment of abdominal infections, complicated skin and skin and soft tissue infections, and community-acquired pneumonia (Ni et al., 2016). The mechanism of action of tigecycline is similar to that of other tetracyclines, which inhibit bacterial protein synthesis by binding to the 30S subunit of the ribosome (Stein and Babinchak, 2013). In recent years, with the prevalence of carbapenem-resistant Enterobacteriaceae (CRE) and motionless bacilli, polymyxin and tigecycline are beginning to be frequently used in clinical treatment (Rudra et al., 2018; Chen et al., 2021). These two drugs have been identified by the World Health Organization as the last lines of defense against infections caused by MDR Gram-negative bacteria (He et al., 2019). With the dissemination of carbapenem resistance gene *bla*_NDM_ and polymyxin resistance gene *mcr*, tigecycline has been the final choice in the treatment of MDR infections (Choi et al., 2020; Huang et al., 2021). However, novel plasmid-mediated tigecycline resistance genes *tet*(X3/X4) in bacteria of human and animal origin were identified in 2019 in China. More importantly, *tet*(X3/X4) genes are located on conjunctive plasmids, which means that this resistance gene is easily transmitted horizontally and has attracted global attention to *tet*(X)-mediated high levels of tigecycline resistance (Li et al., 2020b; Wen et al., 2020).

Plasmids can facilitate bacterial fitness to a variety of environmental conditions by mediating the horizontal transmission of genetic information (Chen et al., 2019; Starkova et al., 2021). Although plasmids have brought many benefits to host bacteria, plasmids as foreign DNA also cause disorders in bacterial regulatory networks or energy metabolism, thereby leading to corresponding fitness cost to host bacteria and weakening of their competitiveness (San Millan and Maclean, 2017; Pi et al., 2020). Previous studies showed that *mcr*-conferred polymyxin resistance in *Klebsiella pneumoniae* and *Escherichia coli* resulted in a significant biological fitness cost (Yang et al., 2017; Nang et al., 2018). Consequently, bacteria carrying high-cost plasmids will soon be replaced by plasmid-free bacteria without selection pressure; conversely, bacteria bearing plasmids with lower cost are able to persist in the population for a longer period of time and contribute to the spread of drug resistance (Dimitriu et al., 2019; Moghimi et al., 2021). For example, studies have shown that the type of IncI2 plasmids has the lowest fitness cost among the *mcr-1*-bearing plasmids; consistently, epidemiological surveys showed that IncI2 is the most dominant type of *mcr-1*-bearing plasmid, indicating that the fitness cost will affect the prevalence of resistance plasmids (Wu et al., 2018; Pietsch et al., 2021). However, little is known about how *tet*(X4)-bearing plasmids adapt to host bacteria and maintain the resistance genes in the absence and presence of antibiotics (Xu et al., 2021b; Yang et al., 2021).

In this study, we performed a large-scale fitness analysis of different types of *tet*(X4)-positive plasmids including IncX1, IncFIA, IncFIB, IncA/C2 (IncA), IncQ1, and IncFII in a model strain *E. coli* TOP10. Furthermore, we investigated the fitness cost of representative *tet*(X4)-bearing plasmids in the presence of different classes of antibiotics. Our results advance the understanding of the evolutionary trajectory of the *tet*(X4) gene in pathogens and provide basic information for its transmission characteristics and epidemic trend.

## Materials and Methods

### Bacterial Strains, Plasmids, and Reagents

All clinical strains used in the study were collected from a slaughterhouse in Jiangsu Province, China, and preserved in our laboratory (Li et al., 2020a). The strain and plasmid information is shown in **Table S1**. These *tet*(X4) plasmids were extracted and electroporated into a reference strain *E. coli* TOP10. These transformants were cultured on selective LB agar (Qingdao Hope Bio-Technology, China) with tigecycline (2 μg/ml) at 37°C, and the success of plasmid electroporation was verified by PCR analysis. All chemical reagents were purchased from Yuanye Bio-Technology Co., Ltd. (Shanghai, China).

### Growth Curve Determination

Five microliters of overnight bacterial culture was diluted into 5 ml LB broth at 37°C with 200 rpm shaking. Then, 200 μl of bacterial culture was added into a 96-well flat-bottom plate every hour during the culture process; blank LB broth was used as a negative control. Growth curves were determined by measuring the optical density at 620 nm using a spectrophotometer (Thermo Fisher, Waltham, MA, USA). The aliquots were collected from 0 to 12 h at every hour. Experiments were performed with three biological replicates.

### Biofilm Formation Assay

The crystal violet method was used to assess the ability of biofilm formation of transformants. Briefly, overnight bacterial culture was diluted 1:100 into fresh broth. Cultures were incubated until the mid-log phase and inoculated into a 96-well flat-bottom plate at 37°C for 24 h. Then, the cells were washed with phosphate-buffered saline for three times, and a solution of crystal violet (0.1%) was added into each well and incubated at room temperature for 20 min. Thirty-three percent of acetic acid was added into each well in an incubator at 37°C for 30 min as previously described (Cheng et al., 2021). Finally, the absorbance of suspension at 570 nm was determined using an Infinite M200 Microplate reader (Tecan, Männedorf, Switzerland). Experiments were performed with three biological replicates.

### Antimicrobial Susceptibility Testing

The minimum inhibitory concentrations (MICs) of eight antimicrobial agents, namely, polymyxin, tetracycline, oxytetracycline, chlortetracycline, florfenicol, tiamulin, enrofloxacin, and ceftiofur, were determined using the broth microdilution method according to the Clinical and Laboratory Standards Institute (CLSI) guidelines (CLSI, 2018). *E. coli* ATCC25922 was used as a control, and all experiments were performed with three biological replicates.

### *In Vitro* Competition Assay

Transformants and their parental strain TOP10 were selected and cultured into the logarithmic growth phase and mixed in 5 ml LB broth at a ratio of 1:1, then the mixtures were incubated at 37°C with 200 rpm shaking. The mixed bacterial culture at 0, 24, 48, and 72 h was successively diluted 10-fold in normal saline and cultured on LB agar plates with tigecycline overnight at 37°C. The relative fitness of two strains was calculated by the following formula: competition index (CI) = ln (RF/RI)/ln (SF/SI) (Delafuente et al., 2020). RI and SI indicated the number of resistant and sensitive bacteria in the bacterial culture at 0 h, and RF and SF indicated the number of resistant and sensitive bacteria in the bacterial culture at different time points. The competition was plotted with time as abscissa and competition index as ordinate. If CI > 1, it indicates that the fitness of resistant bacteria is stronger than that of sensitive bacteria; if CI < 1, it indicates that the fitness of resistant bacteria is weaker than that of sensitive bacteria; and if CI = 1, it indicates that the competitiveness of the two is equivalent.

In evolution experiments, a single colony on a McConkey agar plate was selected and inoculated into broth with tigecycline, doxycycline, tetracycline, oxytetracycline, chlortetracycline, or ceftiofur. The bacterial culture was cultured at 37°C with 200 rpm shaking. The initial bacterial culture was used as the first passage; the mixtures were transferred into fresh LB broth after a 12-h culture and serve as the second passage. The bacterial culture of every 10 passages was stored with 40% glycerol at -80°C.

### Virulence Testing

The virulence of strains was tested using a *Galleria mellonella* infection model (Huiyude Biotech Company, Tianjin, China). The experimental strains were cultured at 37°C with 200 rpm for 6 h, and the bacterial culture was adjusted with an OD_620_ of 0.5. The larvae in good growth status were selected and resuscitated at 37°C for 2 h. Larvae (n = 8 per group) were infected with 10 μl bacterial suspension (10^5^ CFUs) at the right posterior gastropoda using a microsyringe. Normal saline was used as a negative control, and the survival of larvae was monitored during 5 days.

### Plasmid Stability Assay

The plasmid stability of transconjugants was determined based on a previous study (Li et al., 2021b). Ten microliters of overnight seed cultures of the strains was inoculated into 10 ml antibiotic-free LB medium (1:1,000 ratio). After culture for 1 to 7 days, bacterial numbers were counted by plating onto LB agar plates and tigecycline-containing plates. Experiments were performed with three biological replicates.

### Statistical Analyses

Data are presented as mean ± SD and analyzed by GraphPad Prism version 8.3.0. Statistical differences were assessed using Student’s *t*-test or one-way ANOVA. The significant difference was defined as *P* < 0.05.

## Results

### Characteristics of *tet*(X4)-Encoding Plasmids and Plasmid Stability

Twenty *tet*(X4)-positive plasmids from our previous study (Li et al., 2020a) were chosen in this study (**Table S1**). These *tet*(X4)-bearing plasmids ranged from 50 to 398 kb and were designated as IncFII, IncFIA, IncFIB, IncX, IncA, and IncQ1 with only one replicon. Meanwhile, these *tet*(X4)-bearing elements could be successfully transferred into the recipient strains, suggesting that these plasmids are conjugative. To investigate the impact of *tet*(X4)-encoding plasmids on host bacteria and avoid the influence of bacterial genome differences, these *tet*(X4)-bearing plasmids were transferred into a reference strain *E. coli* TOP10. Subsequently, we tested the antibiotic susceptibility of these transformants carrying *tet*(X4) plasmids. Results showed that all strains showed high-level resistance to all tetracyclines, including tigecycline (**Table S2**), suggesting the successful expression of the *tet*(X4) gene in TOP10. Furthermore, most *tet*(X4)-bearing strains conferred multiple resistance to other classes of antibiotics such as florfenicol and tiamulin, indicating that these plasmids carried various antibiotic resistance genes.

Next, we investigated the stability of these *tet*(X4)-positive plasmids in transformants in the antibiotic-free LB broth (**Figure S1**). Consequently, we found that the loss rate of most of *tet*(X4)-bearing plasmids was less than 50% over 7 days without the pressure of tigecycline, implying that the *tet*(X4) gene can be moderately maintained by transformants. In comparison, IncFII plasmid-carrying transformants displayed the minimal deletion of plasmids, indicating that IncFII plasmids may be the important vector for the transmission of *tet*(X4).

### Bacterial Growth Comparison of Transformants and TOP10

**Figure 1:**
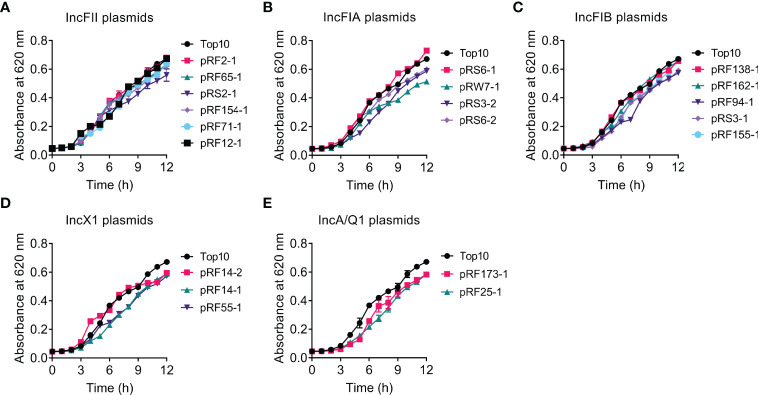
Effects of *tet*(X4)-positive plasmids on the growth rate of transformants. Growth curves of TOP10 and transformants carrying *tet*(X)-positive IncFII **(A)**, IncFIA **(B)**, IncFIB **(C)**, IncX1 **(D)**, or IncA/Q1 **(E)** plasmids during 12 h. Y axis, absorbance of bacterial cultures at 620 nm; X axis, time of bacteria growth (hours).

To investigate whether *tet*(X4)-bearing plasmids would impose a fitness burden to TOP10, we compared the bacterial growth of transformants and their parental strain TOP10 by monitoring the absorbance of culture at 620 nm over 12 h (**Figure 1**). Consequently, the carriage of most *tet*(X4)-positive plasmids delayed or decreased bacterial growth compared with TOP10. In comparison, minimal differences in growth between IncFII plasmid-carrying transformants (except for pRS2-1, 117 kb) and TOP10 after 12 h were found (**Figure 1A**). In addition, the introduction of pRS6-1 (IncFIA, 138 kb), pRF138-1 (IncFIB, 140 kb), and pRF162-1 (IncFIB, 138 kb) had no significant effect on the growth of TOP10. These results indicate that *tet*(X4)-bearing IncFII plasmids have the lowest effect on the growth of recipient bacteria, and the effect on growth does not seem to be directly related to the size of plasmids.

### Effect of *tet*(X4)-Positive Plasmids on Bacterial Fitness

**Figure 2:**
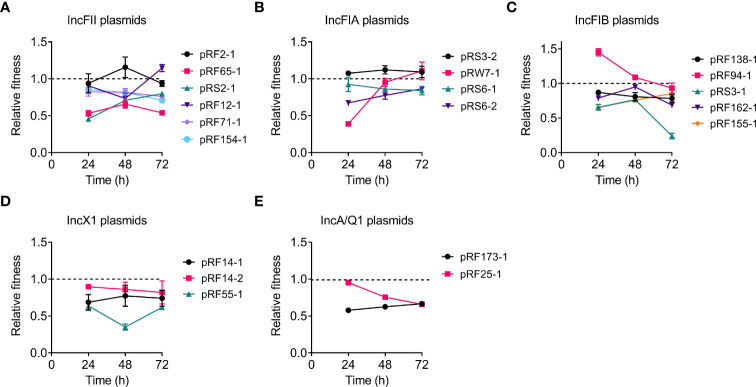
Effects of *tet*(X4)-positive plasmids on the fitness cost of transformants. Transformants carrying *tet*(X)-positive IncFII **(A)**, IncFIA **(B)**, IncFIB **(C)**, IncX1 **(D)**, or IncA/Q1 **(E)** plasmids were competed with their parental strain *E. coli* TOP10, respectively. The relative fitness of transformants was detected every day (from 24 to 72 h). All competition assays were performed with three biological replicates.

To get a more comprehensive understanding of the fitness cost induced by *tet*(X4)-positive plasmids, we investigated whether *tet*(X4)-bearing plasmids would impose a fitness defect to the plasmid-free *E. coli* TOP10 using competition experiments. Twenty transformants carrying different types of *tet*(X4)-positive plasmids were competed with the plasmid-free *E. coli* TOP10 in the media without antibiotics, and the ratios of transformants to plasmid-free isolates were determined at 24, 48, and 72 h. Consistent with bacterial growth experiments, competition experiments showed that most of transformants were less competitive than TOP10, suggesting the modest fitness cost by *tet*(X4)-positive plasmids (**Figure 2**). Specifically, most IncFII and IncFIA plasmid-carrying transformants showed comparable competitiveness against TOP10 at 72 h, but all IncFIB, IncX1, IncA, and IncQ1 plasmids showed CI values of less than 1, suggesting that they were less competitive than susceptible strains (**Figures 2A–E**). Interestingly, the fitness advantage of pRW7-1 (IncFIA) was substantially increased after serial passages, with CI values from 0.4 to 1.1. By contrast, the fitness advantage of pRF94-1 (IncFIB) was remarkably reduced after serial passages, with CI values from 1.45 to 0.92. Importantly, two plasmids including pRF2-1 (IncFII) and pRS3-2 (IncFIA) were more competitive than TOP10, indicating that they maintained a high fitness advantage to recipient bacteria. Consistently, these two plasmids had similar growth curves with TOP10. Taken together, these results indicate that *tet*(X4)-positive plasmids impose varying degrees of fitness cost on host bacteria.

### Effects of *tet*(X4)-Positive Plasmids on Bacterial Biofilm Formation

**Figure 3:**
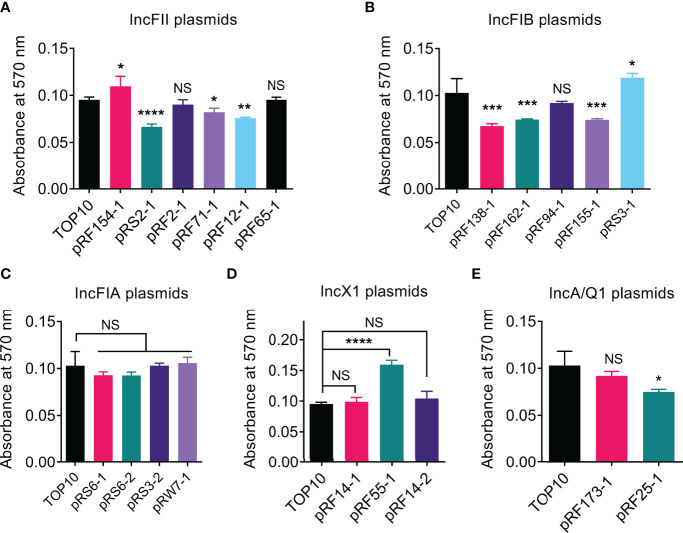
The effect of *tet*(X4)-positive plasmids on biofilm formation. Comparison of biofilm formation between TOP10 and transformants carrying IncFII **(A)**, IncFIB **(B)**, IncFIA **(C)**, IncX1 **(D)**, or IncA/Q1 plasmids **(E)**. Biofilm formation ability was assessed using crystal violet method. Data are shown as mean ± SD from three biological replicates. Statistical analyses were conducted with unpaired, two-tailed *t*-test (*P ≤ 0.05, **P ≤ 0.01, ***P ≤ 0.001, ****P ≤ 0.0001). NS, not significant.

It is suggested that the ability of biofilm formation in bacteria is associated with antibiotic resistance and is a major virulence factor for bacterial pathogenicity (Hall and Mah, 2017; Yan and Bassler, 2019). Thus, we next compared the biofilm formation of transformants carrying *tet*(X4)-positive plasmids and its parental strain TOP10 using crystal violet assay. Interestingly, we found that the carriage of these *tet*(X4)-positive plasmids led to a heterogeneous effect on the ability of biofilm formation in the recipient. Among 20 *tet*(X4)-positive plasmids, three plasmids, namely, pRF154-1 (IncFII), pRS3-1 (IncFIB), and pRF55-1 (IncX1), significantly promoted the biofilm production, whereas seven plasmids belonging to IncFII (3), IncFIB (3), and IncQ1 (1), respectively, markedly decreased the biofilm formation of host bacteria (**Figure 3**). Other 10 *tet*(X4)-bearing plasmids including all IncFIA types had no effect on biofilm production.

### Effects of *tet*(X4)-Positive Plasmids on Bacterial Virulence

**Figure 4:**
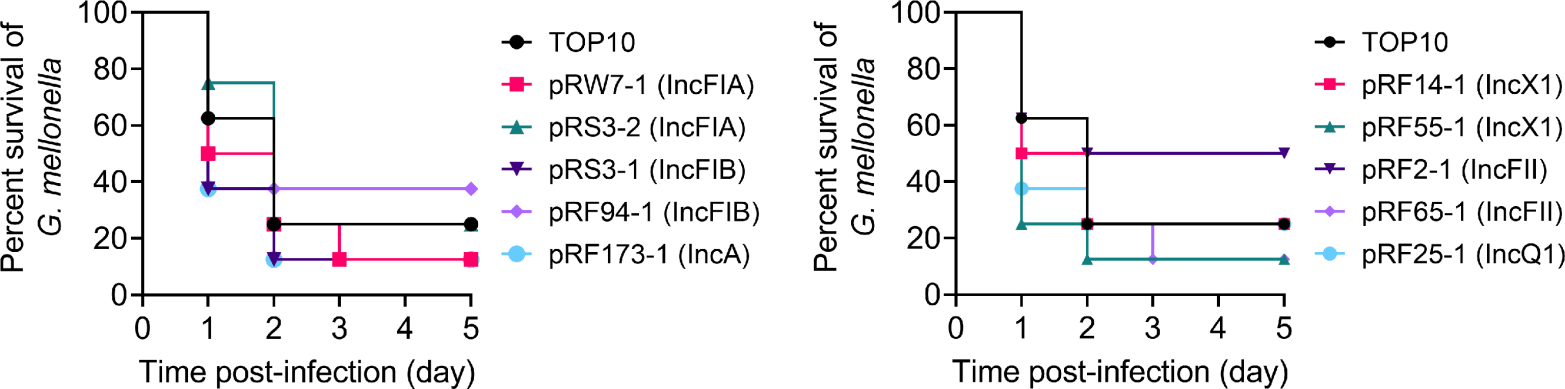
Survival of *G. mellonella* larvae after infection with *E. coli* TOP10 and transformants carrying different types of *tet*(X4)-positive plasmids after 5 days postinfection.

Bacterial pathogenicity is an important factor to assess the fitness cost; 10 representative strains were chosen for further virulence experiments. A *G. mellonella* larval infection model was further applied to explore the impact of different types of *tet*(X4) plasmids on the pathogenicity of TOP10. We found that all transformants resulted in a time-dependent killing of larvae during 5 days (**Figure 4**). Some transformants with a single plasmid showed increased *G. mellonella* larval killing ability compared to the plasmid-free recipient strain TOP10. For example, the survival of *G. mellonella* larvae infected with IncFIA and IncX1 plasmid-carrying strains was less than 20% after 2 days, suggesting that these transformants showed an increased virulence. By contrast, the survival rate of *G. mellonella* larvae infected with pRF94-1 (IncFIB) and pRF2-1 (IncFII) plasmid-carrying host bacteria was higher than that of TOP10, indicating that the carriage of these two plasmids reduced the virulence of host bacteria. These data indicate that the introduction of *tet*(X4)-positive plasmids may affect the virulence of host bacteria by regulating bacterial fitness.

### Fitness Cost of Transformants in the Absence or Presence of Antibiotics

**Figure 5:**
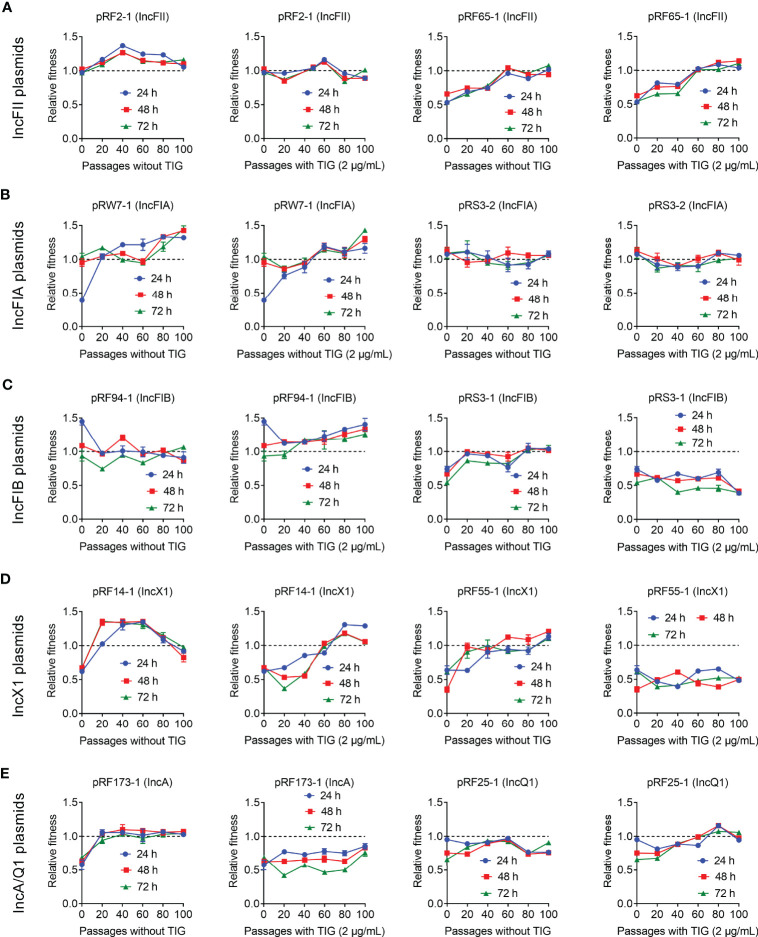
Fitness cost changes of *tet*(X4)-bearing plasmids in the absence or presence of tigecycline. Transformants carrying *tet*(X)-positive IncFII **(A)**, IncFIA **(B)**, IncFIB **(C)**, IncX1 **(D)**, and IncA/Q1 **(E)** plasmids were competed with a reference strain *E. coli* TOP10, respectively. All competitions assays were carried out with three biological replicates and last for 100 passages.

To better understand the impact of passage evolution on the fitness of *tet*(X4)-bearing strains, 10 representative transformants harboring different types of plasmids were selected and continuously cultured in the absence or presence of tigecycline for 100 passages. Meanwhile, the relative fitness of these transformants at 20, 40, 60, 80, and 100 passages was determined by competing with plasmid-free TOP10. As a consequence, the fitness advantages of most transformants were maintained or improved after serial passages without or with tigecycline (**Figure 5**). Interestingly, the serial passages under tigecycline had no effect on the fitness of three plasmids, namely, pRF55-1 (IncX1), pRS3-1 (IncFIB), and pRF173-1 (IncA), which still imposed high fitness cost on TOP10 in the passages. These results indicate that the direct pressure of tigecycline is not necessary for the fitness evolution of some *tet*(X4)-positive plasmids.

**Figure 6:**
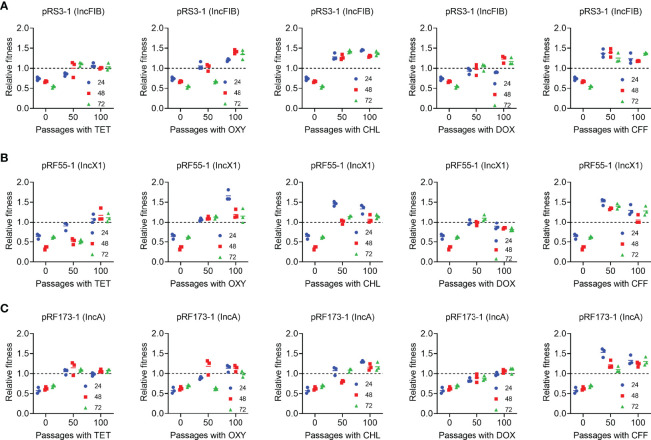
Fitness cost changes of three transformants carrying different types of *tet*(X4)-bearing plasmids in the presence of five commonly used drugs in clinic involve tetracycline (TET), oxytetracycline (OXY), chlortetracycline (CHL), doxycycline (DOX), and ceftiofur (CFF). *In vitro* competitive experiments of three transformants including pRS3-1 [IncFIB, **(A)**], pRF55-1 [IncX1, **(B)**], and pRF173-1 [IncA, **(C)**], which were competed with a reference strain *E. coil* TOP10 under the pressure of five drugs and last for 100 passages. The relative fitness was calculated at 24, 48, and 72 h.

Considering that tigecycline as a critical human medicine has not been employed in animal husbandry, we next sought to explore whether other commonly used veterinary drugs can promote the evolution of *tet*(X4)-positive bacteria and improve their fitness advantages. To this end, four tetracyclines (tetracycline, oxytetracycline, chlortetracycline, and doxycycline) and ceftiofur were chosen for the following evolution experiments (**Figure 6**). Three selected transformants, namely, pRF55-1 (IncX1), pRS3-1 (IncFIB), and pRF173-1 (IncA), were passaged for 50 days (a total of 100 passages) under the exposure of five antibiotics. Interestingly, we found that all strains showed improved fitness after experimental evolution under the pressure of five drugs.

Furthermore, we evaluated the plasmid stability of evolved *tet*(X4)-bearing strains. Specifically, strains carrying plasmids were passaged for 50 days (a total of 100 passages) with tigecycline pressure and serially passaged in drug-free broth for 10 days (20 passages in total), then the loss rate of plasmids was measured. In the absence of tigecycline, the loss rate of plasmids in evolved strains was less than 20% in most strains, except for three strains, namely, pRF65-1, pRF173-1, and pRF25-1 (**Figure S2**). It is worth noting that plasmid pRF2-1 (IncFII) had the best stability, with a loss rate of less than 5%. In comparison, the evolved strains had higher plasmid stability than their parental strains. These results imply that antibiotic pressure can decrease the fitness cost of *tet*(X4)-carrying plasmids on host bacteria and improve the plasmid stability.

## Discussion

The dissemination of the *tet*(X4) gene in human beings, animals, and environments is an increasing threat to global health. It is suggested that antibiotic resistance plasmids would impose a fitness cost on the hosts, and in general they were not retained in the cell without selective pressure (Kozuch et al., 2020; Gaffke et al., 2021). However, many studies have shown that some plasmids can persist in bacterial populations for a long time, even without positive selection, which is referred to as the “plasmid paradox” (Harrison and Brockhurst, 2012). For example, it is reported that the acquisition of mobile *bla*_NDM-5_-positive plasmids in Enterobacteriaceae or *K. pneumoniae* showed little fitness cost on host bacteria (Ma et al., 2020; Huang et al., 2021). It is plausible that antibiotic resistance is determined by interactions between the resistance gene and bacterial host, not only by the existence of the resistance gene (Praski Alzrigat et al., 2021). San et al. found that a small plasmid pB1000 carrying *bla*_ROB-1_ was able to improve bacterial resistance levels by increasing fitness advantages (San Millan and Maclean, 2017; Sun et al., 2019b). Therefore, understanding the underpinnings of how these *tet*(X4)-positive plasmids adapt to host bacteria and maintain the persistence of resistance genes is of importance for monitoring and controlling the prevalence of the *tet*(X4) gene (Zhou et al., 2021). In this study, we conducted a large-scale analysis of fitness cost elicited by different types of *tet*(X4)-positive plasmids in a reference strain *E. coli* TOP10. Interestingly, we found that although some types of *tet*(X4)-positive plasmids exhibited moderate fitness cost on the host, experimental evolution under antibiotic pressure can significantly improve their adaptability.

The fitness cost of strains is often manifested in reducing bacterial growth rates, competitiveness, or virulence (Sun et al., 2019a). For example, the increased expression of *mcr-1* results in decreased growth rate, cell viability, and attenuated virulence (Yang et al., 2017). Consistently, the introduction of most *tet*(X4)-positive plasmids mildly decreased the growth rate and relative fitness of host bacteria compared with its parental strain TOP10. Interestingly, some types of *tet*(X4)-positive plasmids particularly for IncFII plasmids had little effect on the bacterial growth and fitness. In addition, we evaluated the impact of these *tet*(X4)-plasmids on bacterial biofilm formation and virulence in a *G. mellonella* infection model, which can help us to better understand the fitness cost caused by the resistance plasmids. In agreement with the fitness results, the carriage of *tet*(X4)-positive plasmids modestly reduced the biofilm production and improved the survival rate of larvae, suggesting that the biofilm formation ability was connected with bacterial virulence. However, three plasmids, namely, pRF154-1 (IncFII), pRS3-1 (IncFIB), and pRF55-1 (IncX1), significantly promoted the biofilm formation and resulted in high mortality on larvae. These results were interesting and inconsistent with most of ARGs. For example, it is suggested that high-level colistin resistance mutants (HLCRMs) with high expression of *mcr-1* markedly attenuated virulence in a *G. mellonella* infection model (Yang et al., 2017). Similar to these three plasmids, it is reported that the biofilm formation ability of bacteria was enhanced by carrying the p3R-IncX3-type plasmid of *bla*_NDM-5_, which may accelerate the spread of the *bla*_NDM-5_ gene (Ma et al., 2020). We speculated that these plasmids may carry virulence genes or corresponding virulence regulators, which enhanced the pathogenicity of host bacteria. Furthermore, we investigated the fitness cost of *tet*(X4)-positive bacteria after serial passages in the presence of tigecycline or other antibiotics. Importantly, we found that the fitness advantage of most evolved strains was generally improved by experimental evolution, accompanied by the increased plasmid stability. Tigecycline is currently only used in human clinical practice, but *tet*(X4)-positive strains have been widely found in aquaculture environments (Babaei and Haeili, 2021; Fu et al., 2021). In contrast, other tetracyclines such as tetracycline, oxytetracycline, chlortetracycline, and doxycycline are commonly used in veterinary clinics in China (Li et al., 2021a; Nji et al., 2021). Consistent with this notion, some high fitness cost strains even in the presence of tigecycline showed improved fitness advantages under the exposure of other commonly used veterinary medicines. These data imply that the pressure of tigecycline is not necessary for the maintenance of the *tet*(X4) gene, and the widespread prevalence of the *tet*(X4) resistance gene may be associated with the use of other tetracyclines. We reasoned that there are some regulation mechanisms or compensatory evolution in this process, which needs to be further explored.

To conclude, our study indicates that the introduction of most of *tet*(X4)-harboring plasmids can impose varying degrees of fitness cost on host bacteria, but the fitness cost can be quickly alleviated during the evolution, which may be responsible for the epidemic spread of dominant plasmids in the clinical setting. In addition, the specific genes located in every plasmid may also affect the traits of host bacteria, which required a more comprehensive investigation.

## Data Availability Statement

The original contributions presented in the study are included in the article/**Supplementary Material**. Further inquiries can be directed to the corresponding authors.

## Author Contributions

YL and ZW designed this study. FT and WC performed all experiments and wrote the draft manuscript. YL, FT, and LJ analyzed the data. All authors contributed to the article and approved the submitted version.

## Funding

This work was supported by the National Natural Science Foundation of China (32002331 and 31872526), Agricultural Science and Technology Independent Innovation Fund of Jiangsu Province (CX(20)3091 and CX(21)2010), China Postdoctoral Science Foundation-funded project (2019M651984 and 2021T140579), A Project Funded by the Priority Academic Program Development of Jiangsu Higher Education Institutions (PAPD), and Young Elite Scientists Sponsorship Program by CAST (2020QNRC001).

## Conflict of Interest

The authors declare that the research was conducted in the absence of any commercial or financial relationships that could be construed as a potential conflict of interest.

## Publisher’s Note

All claims expressed in this article are solely those of the authors and do not necessarily represent those of their affiliated organizations, or those of the publisher, the editors and the reviewers. Any product that may be evaluated in this article, or claim that may be made by its manufacturer, is not guaranteed or endorsed by the publisher.

## References

[B1] Babaei S. Haeili M. (2021). Evaluating the Performance Characteristics of Different Antimicrobial Susceptibility Testing Methodologies for Testing Susceptibility of Gram-Negative Bacteria to Tigecycline. BMC Infect. Dis. 21, 709. doi: 10.1186/s12879-021-06338-7 34315422 PMC8314565

[B2] Chen C. Chen L. Zhang Y. Cui C. Y. Wu X. T. He Q.. (2019). Detection of Chromosome-Mediated *Tet*(X4)-Carrying *Aeromonas Caviae* in a Sewage Sample From a Chicken Farm. J. Antimicrob. Chemother. 74, 3628–3630. doi: 10.1093/jac/dkz387 31511873

[B3] Cheng P. Yang Y. Cao S. Liu H. Li X. Sun J.. (2021). Prevalence and Characteristic of Swine-Origin *Mcr-1*-Positive *Escherichia Coli* in Northeastern China. Front. Microbiol. 12, 712707. doi: 10.3389/fmicb.2021.712707 34354696 PMC8329492

[B4] Chen H. L. Jiang Y. Li M. M. Sun Y. Cao J. M. Zhou C.. (2021). Acquisition of Tigecycline Resistance by Carbapenem-Resistant *Klebsiella Pneumoniae* Confers Collateral Hypersensitivity to Aminoglycosides. Front. Microbiol. 12, 674502. doi: 10.3389/fmicb.2021.674502 34276606 PMC8284424

[B5] Choi Y. Lee J. Y. Lee H. Park M. Kang K. Lim S. K.. (2020). Comparison of Fitness Cost and Virulence in Chromosome- and Plasmid-Mediated Colistin-Resistant *Escherichia Coli* . Front. Microbiol. 11, 798. doi: 10.3389/fmicb.2020.00798 32477288 PMC7238749

[B6] Delafuente J. Rodriguez-Beltran J. San Millan A. (2020). Methods to Study Fitness and Compensatory Adaptation in Plasmid-Carrying Bacteria. Methods Mol. Biol. 2075, 371–382. doi: 10.1007/978-1-4939-9877-7_26 31584176

[B7] Dimitriu T. Medaney F. Amanatidou E. Forsyth J. Ellis R. J. Raymond B. (2019). Negative Frequency Dependent Selection on Plasmid Carriage and Low Fitness Costs Maintain Extended Spectrum Beta-Lactamases in *Escherichia Coli* . Sci. Rep. 9, 17211. doi: 10.1038/s41598-019-53575-7 31748602 PMC6868128

[B8] Fang L. X. Chen C. Cui C. Y. Li X. P. Zhang Y. Liao X. P.. (2020). Emerging High-Level Tigecycline Resistance: Novel Tetracycline Destructases Spread *via* the Mobile Tet(X). Bioessays 42, e2000014. doi: 10.1002/bies.202000014 32567703

[B9] Fu Y. Chen Y. Liu D. Yang D. Liu Z. Wang Y.. (2021). Abundance of Tigecycline Resistance Genes and Association With Antibiotic Residues in Chinese Livestock Farms. J. Hazard. Mater. 409, 124921. doi: 10.1016/j.jhazmat.2020.124921 33421874

[B10] Gaffke L. Kubiak K. Cyske Z. Wegrzyn G. (2021). Differential Chromosome- and Plasmid-Borne Resistance of *Escherichia Coli* Hfq Mutants to High Concentrations of Various Antibiotics. Int. J. Mol. Sci. 22, 8886. doi: 10.3390/ijms22168886 34445592 PMC8396180

[B11] Hall C. W. Mah T.-F. (2017). Molecular Mechanisms of Biofilm-Based Antibiotic Resistance and Tolerance in Pathogenic Bacteria. FEMS Microbiol. Rev. 41, 276–301. doi: 10.1093/femsre/fux010 28369412

[B12] Harrison E. Brockhurst M. A. (2012). Plasmid-Mediated Horizontal Gene Transfer is a Coevolutionary Process. Trends Microbiol. 20, 262–267. doi: 10.1016/j.tim.2012.04.003 22564249

[B13] He T. Wang R. Liu D. Walsh T. R. Zhang R. Lv Y.. (2019). Emergence of Plasmid-Mediated High-Level Tigecycline Resistance Genes in Animals and Humans. Nat. Microbiol. 4, 1450–1456. doi: 10.1038/s41564-019-0445-2 31133751

[B14] Huang J. Zhang S. Zhao Z. Chen M. Cao Y. Li B. (2021). Acquisition of a Stable and Transferable *bla*_NDM_*-*_5_-Positive Plasmid With Low Fitness Cost Leading to Ceftazidime/Avibactam Resistance in KPC-2-Producing Klebsiella Pneumoniae During Treatment. Front. Cell. Infect. Microbiol. 11, 658070. doi: 10.3389/fcimb.2021.658070 34354959 PMC8329419

[B15] Kozuch B. C. Shaffer M. G. Culyba M. J. (2020). The Parameter-Fitness Landscape of lexA Autoregulation in *Escherichia Coli* . mSphere 5, e00718–e00720. doi: 10.1128/mSphere.00718-20 32817380 PMC7440846

[B16] Labreck P. T. Merrell D. S. (2020). Fitness Costs Associated With Carriage of a Large Staphylococcal Plasmid are Reduced by Subinhibitory Concentrations of Antiseptics. Microbiologyopen 9, e1005. doi: 10.1002/mbo3.1005 32053737 PMC7142362

[B17] Li W. Liu Z. Yin W. Yang L. Qiao L. Song S.. (2021b). MCR Expression Conferring Varied Fitness Costs on Host Bacteria and Affecting Bacteria Virulence. Antibiotics 10, 872. doi: 10.3390/antibiotics10070872 34356793 PMC8300855

[B18] Li R. Lu X. Peng K. Liu Z. Li Y. Liu Y.. (2020a). Deciphering the Structural Diversity and Classification of the Mobile Tigecycline Resistance Gene Tet(X)-Bearing Plasmidome Among Bacteria. mSystems 5, e00134–e00120. doi: 10.1128/mSystems.00134-20 32345737 PMC7190383

[B19] Li R. Mohsin M. Lu X. Abdullah S. Munir A. Wang Z. (2021a). Emergence of Plasmid-Mediated Resistance Genes Tet(X) and Mcr-1 in *Escherichia Coli* Clinical Isolates From Pakistan. mSphere 6, e0069521. doi: 10.1128/mSphere.00695-21 34431695 PMC8386413

[B20] Li Y. Wang Q. Peng K. Liu Y. Li R. Wang Z. (2020b). Emergence of Carbapenem- and Tigecycline-Resistant Proteus Cibarius of Animal Origin. Front. Microbiol. 11, 1940. doi: 10.3389/fmicb.2020.01940 32922378 PMC7457074

[B21] Ma T. Fu J. Xie N. Ma S. Lei L. Zhai W.. (2020). Fitness Cost of *bla*_NDM_*-*_5_-Carrying P3r-IncX3 Plasmids in Wild-Type NDM-Free Enterobacteriaceae. Microorganisms 8, 377. doi: 10.3390/microorganisms8030377 PMC7143814 32156014

[B22] Moghimi M. Haeili M. Mohajjel Shoja H. (2021). Characterization of Tigecycline Resistance Among Tigecycline Non-Susceptible *Klebsiella Pneumoniae* Isolates From Humans, Food-Producing Animals, and *In Vitro* Selection Assay. Front. Microbiol. 12, 702006. doi: 10.3389/fmicb.2021.702006 34421858 PMC8374936

[B23] Nang S. C. Morris F. C. Mcdonald M. J. Han M. L. Wang J. Strugnell R. A.. (2018). Fitness Cost of *Mcr-1*-Mediated Polymyxin Resistance in *Klebsiella Pneumoniae* . J. Antimicrob. Chemother. 73, 1604–1610. doi: 10.1093/jac/dky061 29514208 PMC6693033

[B24] Ni W. Han Y. Zhao J. Wei C. Cui J. Wang R.. (2016). Tigecycline Treatment Experience Against Multidrug-Resistant *Acinetobacter Baumannii* Infections: A Systematic Review and Meta-Analysis. Int. J. Antimicrob. Agents 47, 107–116. doi: 10.1016/j.ijantimicag.2015.11.011 26742726

[B25] Nji E. Kazibwe J. Hambridge T. Joko C. A. Larbi A. A. Damptey L.. (2021). High Prevalence of Antibiotic Resistance in Commensal *Escherichia Coli* From Healthy Human Sources in Community Settings. Sci. Rep. 11, 3372. doi: 10.1038/s41598-021-82683-4 33564047 PMC7873077

[B26] Pietsch M. Pfeifer Y. Fuchs S. Werner G. (2021). Genome-Based Analyses of Fitness Effects and Compensatory Changes Associated With Acquisition of *bla*_CMY_*-*, *bla*_CTX_*-*_M-_, and *bla*_OXA_*-*_48/VIM-1_-Containing Plasmids in *Escherichia Coli* . Antibiotics 10, 90. doi: 10.3390/antibiotics10010090 33477799 PMC7832316

[B27] Pi R. Liu Q. Takiff H. E. Gao Q. (2020). Fitness Cost and Compensatory Evolution in Levofloxacin-Resistant *Mycobacterium Aurum* . Antimicrob. Agents Chemother. 64, e00224–e00220. doi: 10.1128/AAC.00224-20 32482677 PMC7526816

[B28] Praski Alzrigat L. Huseby D. L. Brandis G. Hughes D. (2021). Resistance/fitness Trade-Off is a Barrier to the Evolution of MarR Inactivation Mutants in *Escherichia Coli* . J. Antimicrob. Chemother. 76, 77–83. doi: 10.1093/jac/dkaa417 33089314 PMC7729382

[B29] Rudra P. Hurst-Hess K. Lappierre P. Ghosh P. (2018). High Levels of Intrinsic Tetracycline Resistance in *Mycobacterium Abscessus* are Conferred by a Tetracycline-Modifying Monooxygenase. Antimicrob. Agents Chemother. 62, e00119–e00118. doi: 10.1128/AAC.00119-18 29632012 PMC5971581

[B30] San Millan A. Maclean R. C. (2017). Fitness Costs of Plasmids: A Limit to Plasmid Transmission. Microbiol. Spectr. 5, MTBP–0016-2017. doi: 10.1128/microbiolspec.MTBP-0016-2017 PMC11687550 28944751

[B31] Starkova P. Lazareva I. Avdeeva A. Sulian O. Likholetova D. Ageevets V.. (2021). Emergence of Hybrid Resistance and Virulence Plasmids Harboring New Delhi Metallo-Beta-Lactamase in *Klebsiella Pneumoniae* in Russia. Antibiotics 10, 691. doi: 10.3390/antibiotics10060691 34207702 PMC8226487

[B32] Stein G. E. Babinchak T. (2013). Tigecycline: An Update. Diagn. Microbiol. Infect. Dis. 75, 331–336. doi: 10.1016/j.diagmicrobio.2012.12.004 23357291

[B33] Sun J. Chen C. Cui C. Y. Zhang Y. Liu X. Cui Z. H.. (2019b). Plasmid-Encoded *Tet*(X) Genes That Confer High-Level Tigecycline Resistance in *Escherichia Coli* . Nat. Microbiol. 4, 1457–1464. doi: 10.1038/s41564-019-0496-4 31235960 PMC6707864

[B34] Sun C. Cui M. Zhang S. Wang H. Song L. Zhang C.. (2019a). Plasmid-Mediated Tigecycline-Resistant Gene Tet (X4) in Escherichia Coli From Food-Producing Animals, China 2008-2018. Emerg. Microbes Infect. 8, 1524–1527. doi: 10.1080/22221751.2019.1678367 31631781 PMC6818123

[B35] Wen X. Huang J. Cao J. Xu J. Mi J. Wang Y.. (2020). Heterologous Expression of the Tetracycline Resistance Gene *tet*X to Enhance Degradability and Safety in Doxycycline Degradation. Ecotoxicol. Environ. Saf. 191, 110214. doi: 10.1016/j.ecoenv.2020.110214 31968275

[B36] Wu R. Yi L. X. Yu L. F. Wang J. Liu Y. Chen X.. (2018). Fitness Advantage of *Mcr-1*-Bearing IncI2 and IncX4 Plasmids *In Vitro* . Front. Microbiol. 9, 331. doi: 10.3389/fmicb.2018.00331 29535696 PMC5835064

[B37] Xu Y. Liu L. Zhang H. Feng Y. (2021b). Co-Production of Tet(X) and MCR-1, Two Resistance Enzymes by a Single Plasmid. Environ. Microbiol. 23, 7445–7464. doi: 10.1111/1462-2920.15425 33559156

[B38] Xu J. Zhu Z. Chen Y. Wang W. He F. (2021a). The Plasmid-Borne *Tet*(A) Gene is an Important Factor Causing Tigecycline Resistance in ST11 Carbapenem-Resistant *Klebsiella Pneumoniae* Under Selective Pressure. Front. Microbiol. 12, 644949. doi: 10.3389/fmicb.2021.644949 33717043 PMC7943888

[B39] Yan J. Bassler B. L. (2019). Surviving as a Community: Antibiotic Tolerance and Persistence in Bacterial Biofilms. Cell Host Microbe 26, 15–21. doi: 10.1016/j.chom.2019.06.002 31295420 PMC6629468

[B40] Yang Q. Li M. Spiller O. B. Andrey D. O. Hinchliffe P. Li H.. (2017). Balancing *Mcr-1* Expression and Bacterial Survival is a Delicate Equilibrium Between Essential Cellular Defence Mechanisms. Nat. Commun. 8, 2054. doi: 10.1038/s41467-017-02149-0 29233990 PMC5727292

[B41] Yang J. Wang H. H. Lu Y. Yi L. X. Deng Y. Lv L.. (2021). A ProQ/FinO Family Protein Involved in Plasmid Copy Number Control Favours Fitness of Bacteria Carrying Mcr-1-Bearing IncI2 Plasmids. Nucleic Acids Res. 49, 3981–3996. doi: 10.1093/nar/gkab149 33721023 PMC8053102

[B42] Zhong X. Xu H. Chen D. Zhou H. Hu X. Cheng G. (2014). First Emergence of *acrAB* and *oqxAB* Mediated Tigecycline Resistance in Clinical Isolates of *Klebsiella Pneumoniae* Pre-Dating the Use of Tigecycline in a Chinese Hospital. PLoS One 9, e115185. doi: 10.1371/journal.pone.0115185 25503276 PMC4264890

[B43] Zhou Y. Fang J. Davood Z. Han J. Qu D. (2021). Fitness Cost and Compensation Mechanism of Sulfonamide Resistance Genes (*Sul1, Sul2*, and *Sul3*) in Escherichia Coli. Environ. Microbiol. 23, 7538–7549. doi: 10.1111/1462-2920.15783 34554624

