# Supplementary material for: Large-Scale Analysis of Fitness Cost of tet(X4)-Positive Plasmids in Escherichia coli
Source: Front Cell Infect Microbiol. 2022 Jun 3;12:798802. doi: 10.3389/fcimb.2022.798802 (PMC9203853; doi:10.3389/fcimb.2022.798802)
Supplement: Supplementary file 1 [file DataSheet_1.docx]

**Supplementary materials**

**Figures**





**Figure S1 Plasmid stability of *tet*(X4) plasmid-harboring transformants.**

Stability of *tet*(X4)-positive plasmids involve IncFII (A), IncFIA (B), IncFIB (C), IncX1 (D) and IncA/Q1 (E) plasmids in transformants over 7 days. Plasmid stability was determined by colony picking onto selective media in plates and presented as percentage of bacterial growth on media with tigecycline.





**Figure S2 Plasmid stability of *tet*(X4)-harboring evolved strains.**

The strains carrying plasmids were passaged for 50 days (a total of 100 generations) with tigecycline pressure, and serially passaged in drug-free broth for 10 days (20 generations in total), then the loss rate of plasmids was measured to determine their stability.

**Tables**

**Table S1 Genetic features of 20 *tet*(X4)-positive plasmids.**

| Plasmids | Size(kb) | Conjugative | Inc-type | *tet*(X4) repeats |
| --- | --- | --- | --- | --- |
| pRF14-1_50k_tetX | 50 | + | IncX1 | one |
| pRF55-1_130k_tetX | 130 | + |  | one |
| pRF14-2_170k_tetX_flye | 170 | + |  | one |
| pRF138-140k_tetX | 140 | + | IncFIB | one |
| pRF162-1_138k_tetX_flye | 138 | + |  | one |
| pRF94-1_170k_tetX | 170 | + |  | one |
| pRF155-1_244k_tetX | 244 | + |  | one |
| pRS3-1_136k_tetX_flye | 136 | + |  | one |
| pRF173-1_110k_tetX | 110 | + | IncA/C2 | one |
| pRF25-1_128k_tetX_flye | 128 | + | IncQ1 | one |
| pRS6-1_138k_tetX_flye | 138 | + | IncFIA | one |
| pRS6-2_244k_tetX_flye | 244 | + |  | one |
| pRS3-2_136k_tetX_flye | 136 | + |  | one |
| pRW7-1_194k_tetX_flye | 194 | + |  | one |
| pRF154-1_398k_tetX_flye | 398 | + | IncFⅡ | one |
| pRF2-1_117k_tetX_flye | 117 | + |  | one |
| pRF65_1_113k_tetX_flye | 113 | + |  | one |
| pRF71-1_112k_tetX_flye | 112 | + |  | one |
| pRS2-1_310k_tetX_flye | 310 | + |  | one |
| pRF12-1_216k_tetX_flye | 216 | + |  | one |

**Table S2 Antimicrobial resistance profile of *E. coli* strains used in this study.**

| Strain/drug | TET | OXY | FFC | CFF | ENR | CHL | TAM | TIG |
| --- | --- | --- | --- | --- | --- | --- | --- | --- |
| ATCC25922 | 0.5 | 2 | 8 | 0.5 | ≤0.25 | 1 | 32 | ≤0.25 |
| TOP10 | 8 | 4 | 4 | 1 | ≤0.25 | 8 | 2 | 2 |
| +pRF65-1 | >128 | 64 | 8 | 1 | ≤0.25 | 64 | >128 | 32 |
| +pRF2-1 | 64 | 16 | 64 | 0.5 | 2 | 64 | >128 | 16 |
| +pRW7-1 | 32 | 4 | 8 | 1 | ≤0.25 | 8 | 128 | 16 |
| +pRS3-2 | 64 | 32 | 16 | 8 | 32 | 16 | >128 | 8 |
| +pRF94-1 | 64 | 64 | 32 | 1 | 32 | 64 | 128 | 32 |
| +pRS3-1 | 32 | 128 | 16 | 8 | 1 | 64 | 2 | 16 |
| +pRF14-1 | 128 | 32 | 4 | 2 | ≤0.25 | 32 | >128 | 16 |
| +pRF55-1 | 32 | 128 | 16 | 8 | 0.5 | 64 | 1 | 32 |
| +pRF25-1 | 64 | 32 | 8 | 1 | ≤0.25 | 32 | >128 | 16 |
| +pRF173-1 | >128 | 64 | 8 | 0.5 | ≤0.25 | 128 | >128 | 16 |

TET, tetracycline; OXY, oxytetracycline; FFC, florfenicol; CFF, ceftiofur; ENR, enrofloxacin; CHL, chlortetracycline; TAM, tiamulin; TIG, tigecycline
